# CD8^+^ T cell exhaustion in the tumor microenvironment of breast cancer

**DOI:** 10.3389/fimmu.2024.1507283

**Published:** 2024-12-09

**Authors:** Hanghang Xie, Xiaowei Xi, Ting Lei, Hongli Liu, Zhijia Xia

**Affiliations:** ^1^ Xi’an People’s Hospital (Xi’an Fourth Hospital), Affiliated People’s Hospital of Northwest University, Xi’an, China; ^2^ Technical University of Munich (TUM) School of Medicine and Health, Munich, Germany; ^3^ Department of General, Visceral, and Transplant Surgery, Ludwig-Maximilians University, Munich, Germany

**Keywords:** CD8, T cell exhaustion, breast cancer, tumor microenvironment, immunotherapy

## Abstract

CD8+ T cells are crucial cytotoxic components of the tumor immune system. In chronic inflammation, they become low-responsive, a state known as T cell exhaustion (TEX). The aim of immune checkpoint blockade is to counteract TEX, yet its dynamics in breast cancer remain poorly understood. This review defines CD8+ TEX and outlines its features and underlying mechanisms. It also discusses the primary mechanisms of CD8+ TEX in breast cancer, covering inhibitory receptors, immunosuppressive cells, cytokines, transcriptomic and epigenetic alterations, metabolic reprogramming, and exosome pathways, offering insights into potential immunotherapy strategies for breast cancer.

## 1 Introduction

Breast cancer (BC) is a prevalent cancer in women worldwide, with its mechanisms not yet fully understood (1). Immune cells play a crucial role from immunosurveillance in normal breast tissue to the progression of BC, including both primary and metastatic stages. The tumor microenvironment in BC shows an increase in immune cells like CD4+ and CD8+ granzyme B+ cytotoxic T cells, B cells, macrophages, and dendritic cells (DCs) (2). In estrogen receptor (ER)-positive tumors, neutrophils and natural killer (NK) cells are most positively associated, while resting memory T cells and CD8+ T cells are negatively associated. In contrast, ER-negative BC shows strong positive correlations with T regulatory and CD8+ cells, with similar negative correlations as ER-positive cases (3). Notably, even early-stage BC patients display exhaustion in tumor-associated CD8^+^ T cells (4), which are essential for eliminating pathogens and tumors (5). Chronic antigen exposure and inflammation in cancer can lead to CD8^+^ T cell exhaustion or altered differentiation (6), with exhausted T cells showing tumor reactivity and proliferation signatures (7). A higher CD8^+^ T cell exhaustion (TEX) score is linked to poorer disease-free survival in BC (8), highlighting the importance of CD8^+^ effector T cell activation versus exhaustion in BC progression and patient outcomes. Additionally, TEX can predict immunotherapy responses in ER-positive BC, underscoring its potential in treatment strategies (9). Recent studies have extensively explored the mechanisms of CD8^+^ TEX in BC. This article summarizes our current understanding of these mechanisms.

## 2 Definition and characteristics of CD8^+^ T cell exhaustion

Tex was first observed in murine models of lymphocytic choriomeningitis virus (LCMV) infection (10). It represents T cell dysfunction with a progressive loss of effector functions during chronic infections and cancer (11). Tex cells exhibit elevated inhibitory receptors (PD-1, CTLA-4, TIM-3, TIGIT, LAG-3), reduced antitumor cytokines (IFN-γ, IL-2, TNF) (6, 11, 12), increased tumor-promoting chemokines, altered transcription factors (TCF1, T-bet, TOX), metabolic issues, and decreased proliferation and survival (6, 11).

In BC, immune cell distribution varies by tumor subtype and between stromal and parenchymal regions (2). CD8+ Tex are present in certain ER+ and triple-negative breast cancers (TNBC), creating a unique tumor microenvironment (TME) with higher IFN-γ activity. Increased CD8+ Tex infiltration is linked to poorer overall and relapse-free survival in premenopausal ER+ BC patients (13). Conversely, TNBC exhibits a more immunosuppressive environment compared to HER2+ or luminal types, with more regulatory T cells (Tregs), exhausted CD8+ T cells, and plasma cells (14).

### 2.1 Mechanism of CD8^+^ T cell exhaustion

Persistent antigen stimulation is essential for inducing T cell exhaustion (15, 16). Additional key signals include pro-inflammatory cytokines (e.g., IFN-α/β, IL-6, IL-27), suppressive cytokines (e.g., IL-10, TGF-β), regulatory leukocytes (e.g., Tregs, immunoregulatory APCs), and TME factors like hypoxia, nutrient deprivation, and altered pH (17–19). Together with chronic TCR engagement, these signals cause sustained upregulation of inhibitory receptors, transcription factor changes, metabolic shifts, and unique transcriptional programs (20–22). This leads to diminished effector functions, altered homeostasis compared to memory T cells, and cell death from overstimulation, resulting in inadequate tumor control (23, 24). Exhausted T cells are regulated by transcriptional and epigenetic mechanisms, including key transcription factors such as NFAT (25), NR4A (26), TOX (27), PTPN2 (28), TCF-1 (29), and Eomes (30). In the BC tumor microenvironment, TEX significantly impacts immune escape and therapeutic response, with immune regulation central to TEX through inhibitory receptors (e.g., PD-1), cytokines (e.g., IL-10), and immunoregulatory cells like TAMs.

## 3 Inhibitory receptors

IRs include PD-1, CTLA-4, *TIGIT*, TIM-3, LAG-3, which are hallmarks of TEX. Exhausted T cells often express high levels of IRs, which act as immunomodulators, limiting immunopathology and promoting tolerance to self-antigens. In BC, the continued expression of inhibitory receptors is critical for TEX.

### 3.1 Programmed death protein 1

PD-1, an inhibitory transmembrane protein on T, B, and NK cells, blocks key T cell pathways (PI3K-AKT-mTOR, RAS-MEK-ERK) when binding PD-L1, leading to T cell exhaustion and conversion to Treg cells (31, 32). Immune checkpoint inhibitors targeting PD-1/PD-L1 are being explored to restore T cell function in BC treatments. In PD-1-high CD8^+^ T cells, miR-149-3p reduces apoptosis and downregulates exhaustion markers by targeting PD-1, TIM-3, BTLA, and Foxp1 mRNAs (33). Additionally, miR-424-5p targets PD-L1 and the PTEN/PI3K/AKT/mTOR pathway, potentially reversing T cell exhaustion (34, 35). BC model DCs overexpress PD-L1 and Gal-9, inducing TEX, but miRNA-5119 mimic-engineered DCs restored function, reduced PD-L1, and boosted immune responses (36). In BC, miRNA-138-5p lowers PD-L1, promoting apoptosis through Caspase-9/3 activation and cell cycle arrest, and affects cell motility and T-cell cytokines by interacting with MMP2, MMP9, vimentin, and E-cadherin (37).

### 3.2 Cytotoxic T lymphocyte-associated protein 4

CTLA-4 (CD152) is a transmembrane protein expressed on regulatory T cells (Tregs), CD4^+^, and CD8^+^ T cells, containing two cytoplasmic domains with tyrosine-based signaling motifs for signal transduction (38). Its extracellular domain, similar to CD28, competitively binds B7-1/2 (CD80/86) on antigen-presenting cells (APCs), modulating T cell function and preventing overactive immune responses (39). Additionally, CTLA-4 is present in the cytoplasm and on the surface of BC cells (40). Elevated serum CTLA-4 in BC patients versus healthy individuals highlights its role in BC pathogenesis and progression (41). Co-culturing CTLA-4^+^ BC cells with human dendritic cells (DCs) inhibits extracellular signal-regulated kinase and activating transcription factor 3 (ATF3), suppressing DC function and maturation (42). These impaired DCs further inhibit the proliferation of allogeneic CD4^+^/CD8^+^ T cells, Th1 differentiation, and cytotoxic T lymphocyte (CTL) function.

### 3.3 Lymphocyte-activation gene 3

Lymphocyte-activation gene 3 (LAG-3 or CD223) is an inhibitory immune checkpoint molecule found on the surface of various lymphocytes (43, 44), including CD4^+^ and CD8^+^ T cells, natural killer (NK) cells, NKT cells, and regulatory T (Treg) cells (45, 46). It shapes the tumor immune environment by inducing T cell exhaustion and limiting proliferation (47). LAG-3 binds ligands like fibrinogen-like protein 1 (FGL1) to suppress T cell activation and is often co-expressed with other checkpoints such as PD-1 or PD-L1 in BC (48–50). Additionally, Liu et al. found that LAG-3 may synergize with multiple immune checkpoints in the BC-induced immune response, including PD-L1, TIGIT, CTLA-4, ICOS, and IDO1 (51).

### 3.4 T-cell Ig and ITIM domain

TIGIT, a member of the immunoglobulin superfamily, is expressed on T lymphocytes and NK cells and is primarily involved in immune responses and inflammatory activities (52). Co-expression of TIGIT with other inhibitory receptors on exhausted CD8^+^ T-cell subsets has been observed in tumors (53). In early-stage breast cancer, high co-expression of TIGIT and other immune checkpoint receptors—including PD-1, CTLA-4, LAG-3, and TIM-3—on tumor-infiltrating lymphocytes correlates with increased disease aggressiveness (54). Elevated levels of TIGIT, TIM-3, and LAG-3 are found in locally advanced breast cancer patients with poor prognostic factors following neoadjuvant chemotherapy (55). TIGIT plays a role in immune and inflammatory responses similar to PD-1 in breast cancer (56), suggesting that TIGIT and PD-1 may synergistically promote the development of severely dysfunctional T cells, as reported in other cancers (57, 58).

### 3.5 T-cell immunoglobulin and mucin-domain containing-3

TIM-3 is a checkpoint receptor expressed on immune cells like dendritic cells, macrophages, and T cells (59–61), mediating immunosuppression through ligands such as phosphatidylserine, CEACAM-1, and galectin-9 (62, 63). In activated T cells, TIM-3 signaling induces exhaustion by decreasing proliferation, reducing effector cytokine production, and promoting apoptosis of cytotoxic T cells (64). In BC, TIM-3 is overexpressed, enhancing tumor cell proliferation, migration, invasion, and inhibiting apoptosis (65–67). High TIM-3 levels correlate with advanced clinical stages, lymph node metastasis, increased Ki67 expression, and poorer 5-year survival rates (65, 66). It promotes tumorigenesis by activating the NF-κB/STAT3 pathway and altering gene expression—upregulating CCND1, C-Myc, MMP1, TWIST, VEGF, and downregulating E-cadherin (68, 69). Additionally, TIM-3 affects tight junction dynamics by downregulating ZO-2, ZO-1, and occludin, which may enhance tumor invasion and migration. Polymorphisms in the TIM-3 gene are also linked to BC susceptibility, progression, and prognosis (70, 71).

Galectin-9 (Gal-9) is an immune checkpoint protein that promotes TEX and modulates the tumor microenvironment (72, 73). As a ligand for TIM-3, Gal-9 induces apoptosis in T cells and suppresses the cytotoxic function of antigen-specific CTLs (74). The TIM-3/Gal-9 pathway is involved in immune evasion by BC cells, which show higher levels of Gal-9 and TIM-3 compared to healthy mammary tissues, with both proteins co-localizing in tumor cells. Upregulation of LPHN2, along with expression of LPHN3 and FLRT3, is also detected in breast tumor cells. Activation of this pathway leads to Gal-9 translocation to the tumor cell surface, protecting them from CTL-induced apoptosis. In patients with TNBC, high Gal-9 expression correlates with positive PD-L1 expression on tumor cells (75).

### 3.6 V-set domain containing T cell activation inhibitor 1

B7-H4 is an inhibitory member of the B7 family and functions as an onco-fetal immune tolerance checkpoint (76, 77). Genetic anomalies in B7-H4 are associated with immune activation and fetal rejection in syngeneic pregnancy models. Similarly, in BC, B7-H4 is linked to tumor progression and correlates with exhaustion of CD8^+^ T cells. Hormonal assays have demonstrated that progesterone induces B7-H4 expression in both placental and BC cells (78).

In summary, exhaustion in CD8^+^ T cells is marked by the concurrent upregulation of multiple immune checkpoints (ICs), with the diversity and quantity of these ICs directly influencing the extent of T cell dysfunction (79–82). Despite the well-documented impacts of individual ICs on T cell functionality, investigations into the additive influence of these ICs on T cell exhaustion in BC remain scant. Nevertheless, compelling data supports the notion that dual IC inhibition, particularly incorporating PD-1, significantly surpasses single IC interventions in boosting tumor-associated CD8^+^ T cell efficacy, both *in vitro* and *in vivo* (83). Besides, nanodrug releasing anti-Galectin-9 antibody can exert local blockade of PD-1/Galectin-9/TIM-3 interaction to enhance effector T cells in BC via reversing the exhaustion (84).

## 4 Immunosuppressive cells and cytokines

In addition to cell surface IRs, immunoregulatory cells and cytokines in the TME—including myeloid-derived suppressor cells (MDSCs), TAMs, and Tregs—contribute to TEX. These immunosuppressive cells hinder immune rejection of malignant cells via cytokine secretion, promoting tumor progression and posing challenges to immunotherapy.

CXCR2^+^ MDSCs inversely correlate with infiltrating CD4^+^ and CD8^+^ T cells. These MDSCs facilitate BC progression and metastasis to lungs and lymph nodes, drive EMT via IL-6, and increase levels of immunosuppressive proteins (PD-1, PD-L1, LAG3, CTLA4, TIM-3) on T cells, contributing to their partial exhaustion mediated by IFN-γ (85).

TAMs are predominant immune cells in BC and can polarize into pro-inflammatory M1 or immunosuppressive M2 phenotypes (86–88). Progranulin (PGRN) promotes CD8^+^ TEX by inducing TAM polarization to M2 macrophages, which suppress T cell proliferation and activation via ICAM-1 interactions (89–91). Elevated CHI3L2 levels in TAMs correlate with poor prognosis in BC. Y-box binding protein 1 (YBX1) is positively associated with M2 macrophage infiltration and TEX markers IDO1 and CTLA4 in luminal BC (92).

IL1R2 in macrophages and BC cells contributes to an immunosuppressive TME. IL1β from TAMs activates IL1R2, increasing PD-L1 levels by promoting YY1 degradation. Inhibiting IL1R2 reduces macrophage recruitment, alters TAM polarization, decreases breast tumor-initiating cell self-renewal, and reduces CD8^+^ TEX (93). Sustained high levels of IL-2 in BC induce CD8^+^ TEX by persistently activating STAT5, which increases tryptophan hydroxylase 1 expression. This enzyme converts tryptophan to 5-hydroxytryptophan (5-HTP), activating the aryl hydrocarbon receptor (AhR) and leading to upregulation of inhibitory receptors and decreased cytokine production, impairing T cell function (94). Low IL-10 expression or loss of IL-10R-STAT3 signaling is linked to increased CD8^+^ TEX and reduced survival in BC patients (95). Conversely, in murine BC models, Heparanase (HPSE)-induced IL-10 upregulation promotes M2 macrophage polarization and TEX (96). Malignant BC cells transfer active TGF-β type II receptor (TβRII) via tumor-derived extracellular vesicles to CD8^+^ T cells, inducing SMAD3 activation and TEX (97). USP8 stabilizes TβRII by deubiquitination, enhancing its expression in plasma membranes and TEVs. USP8 promotes TGF-β/SMAD-induced EMT, invasion, metastasis, and facilitates TβRII^+^ circulating EVs to induce TEX and chemoimmunotherapy resistance (92). RAI14 levels correlate positively with M2 macrophage marker CD163 and TEX marker PD-1, indicating its association with M2 macrophage infiltration and TNBC (98).

## 5 Transcriptomics and epigenetic regulation

Transcriptomic and epigenetic mechanisms play crucial roles in TEX within BC. Epigenetic programming is central to regulating various T cell subsets. In TNBC patients, dysfunctional PD-1^+^ CD8^+^ T cells are enriched with EOMES and nLSD1p. LSD1 promotes TEX by controlling the nuclear localization of EOMES through bivalent post-translational modification at lysine 641, directly affecting T cells (99).

TWIST1, a transcription factor that binds to the PD-L1 promoter and significantly accelerates the exhaustion and death of cytotoxic CD8^+^ T cells in BC (100). Additionally, TWIST1 induces EMT in BC cells via the ITGB1-FAK/ILK signaling pathways and related downstream networks (101). The expression of transcription factor IRF8 facilitates TAM acquisition, antigen presentation of cancer cells, and induction of CTL exhaustion in BC (102). Thymocyte selection-associated high-mobility group box (TOX), involved in TEX during chronic infection and cancer, is associated with better prognosis in BC when highly expressed, as confirmed by survival analysis (103). Bromodomain and extra-terminal (BET) proteins are important in regulating the PD-1/PD-L1 pathway in BC. They mediate interferon-γ (IFN-γ) secretion from activated T cells and IFN-γ signaling in TNBC to induce PD-L1 expression (104).

METTL3 enhances PD-L1 expression in BC cells post-transcriptionally through an m6A-IGF2BP3-dependent mechanism, promoting mRNA stability and affecting the efficacy of tumor immunotherapy (105). Glycosylphosphatidylinositol (GPI) anchor biosynthesis, a common post-translational modification, is elevated in BC patients with severe TEX. Exhausted CD8^+^ T cells exhibit higher levels of GPI anchor biosynthesis than normal CD8^+^ T cells (106).

A comprehensive evaluation of mitochondrial DNA methylation (MTDM) in BC indicates that patients with high MTDM have increased proliferation rates and elevated CD8^+^ TEX, potentially related to the secretion of growth differentiation factor 15 (GDF15) by malignant breast epithelial cells in a high MTDM state (107).

## 6 Metabolic reprogramming

Metabolic reprogramming plays a pivotal role in TEX in BC. Metformin reduces tumor hypoxia and PD-L1 expression, enhances T cell function, and boosts the efficacy of immune checkpoint inhibitors, positioning it as a potential adjunct therapy for refractory tumors like TNBC. At high concentrations, metformin suppresses mTOR signaling and decreases transcription factors (T-bet, Eomes, STAT3), potentially limiting T cell proliferation and cytokine secretion. Notably, metformin reduces PD-1 expression in Jurkat cells and improves the PD-1/CD69 ratio in primary T cells, suggesting a potential to restore T cell activation and counter exhaustion (108).

In mouse BC models, a high-fat diet shifts PD-1^-^ CD8^+^ non-exhausted T cells to PD-1^+^ CD8^+^ exhausted T cells, fostering tumor progression (109). Hypoxia-driven metabolic reprogramming also exacerbates T cell dysfunction and exhaustion. Hypoxia induces HIF1α-dependent epigenetic changes that suppress effector gene expression in T and NK cells, leading to immune dysfunction. Moreover, HIF1α-independent mechanisms promote TEX by upregulating co-inhibitory molecules like TIM-3 or via metabolic stress. Studies show that hypoxia-induced TEX in TNBC models confers resistance to anti-PD1 therapy (110). Additionally, hypoxic stress generates tRNA-derived fragments (tRFs) that bind and displace the 3’ UTR of the oncogenic RNA-binding protein YBX1, inhibiting oncogene stability and suppressing BC progression (111).

## 7 Exosome pathway

Exosomes play a crucial role in intercellular communication, with cancer cell-derived exosomes promoting tumor progression by modulating the tumor microenvironment. Tumor cells secrete PD-L1 via exosomes (exoPD-L1), which binds to PD-1 on CD8^+^ T cells at metastatic sites, inhibiting T cell activation and proliferation, leading to their functional inactivation and immunosuppression (112–115). ICAM1, a glycoprotein involved in immune functions and cancer progression, is secreted in exosomes and regulates the immunosuppressive microenvironment (116). Its interaction with LFA-1 is vital for exoPD-L1 binding to PD-1 (117). Knockdown of ICAM1 in BC cells reduces exosome-induced inhibition of CD8^+^ T cells, suggesting its role in TNBC bone metastasis (116). BC-derived exosomes also suppress CD8^+^ T cell glycolysis via an AKT-mTOR-dependent mechanism, contributing to immune escape (118). In migrating cancer cells, PD-L1 accumulates at the trailing edge, forming migrasomes that can be internalized by adjacent cells, increasing PD-L1 expression and immune suppression, while also releasing chemokines to promote cell migration within the tumor microenvironment (119). The role of migrasomes in CD8^+^ TEX remains understudied.

**Figure 1:**
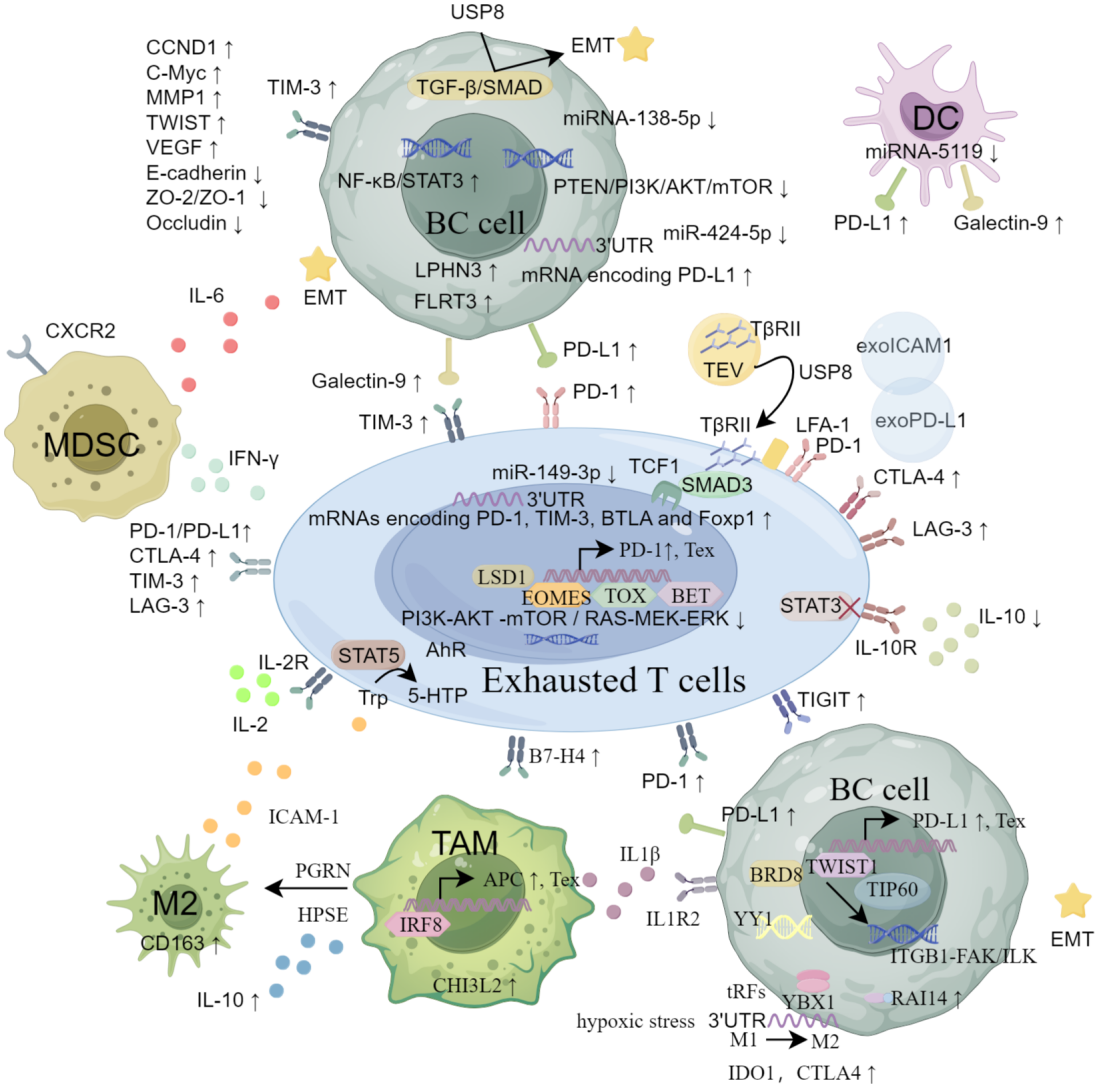
The main mechanisms of CD8^+^ Tex in breast cancer from the aspects of inhibitory receptors, immunosuppressive cells and cytokines, transcriptomics and epigenetic regulation, metabolic reprogramming and exosome pathway.

In summary, TEX is characterized by high expression of IRs, influenced by immune cells and cytokines, with potential mechanisms including transcriptomic, epigenetic, metabolic, and exosome-related pathways (**Figure 1**).

## 8 Discussion

CD8^+^ TEX is critical in immune evasion and progression of BC, involving upregulation of inhibitory receptors (PD-1, CTLA-4, LAG-3, TIM-3), immunosuppressive cells and cytokines, transcriptional and epigenetic alterations, metabolic reprogramming, and exosome pathways. These interactions within the tumor microenvironment diminish T cell function, facilitating tumor growth and metastasis

Understanding these mechanisms is essential for developing effective immunotherapies. While targeting inhibitory receptors with immune checkpoint inhibitors shows promise, their limited efficacy highlights the need for comprehensive approaches. Future research should elucidate molecular pathways of TEX in BC, including specific transcription factors, epigenetic modifications, and metabolic processes. Exploring combination therapies that address multiple aspects of T cell exhaustion may enhance efficacy. Personalizing immunotherapies based on each BC subtype’s unique immune landscape could further optimize patient outcomes. Investigating the role of exosomes and their impact on CD8^+^ T cell function may reveal new therapeutic targets. Integrating these insights can lead to novel interventions to prevent or reverse CD8^+^ TEX, ultimately improving survival and quality of life for BC patients.
